# *rac*-(*OC*-6-33)-bis­[2-(*N*-Benzyl­methyl­imino­meth­yl-κ*N*)-1*H*-imidazol-1-ido-κ*N* ^1^]bis(ethyl­amido)­titanium(IV)

**DOI:** 10.1107/S1600536811013183

**Published:** 2011-04-13

**Authors:** Zhao Li, Wanli Nie, Maxim V. Borzov

**Affiliations:** a Key Laboratory of Synthetic and Natural Chemistry of the Ministry of Education, College of Chemistry and Material Science, the North-West University of Xi’an, Taibai Bei Avenue 229, Xi’an 710069, Shaanxi Province, People’s Republic of China

## Abstract

The title compound, [[Ti(C_2_H_10_N)_2_(C_11_H_10_N_3_)_2_] or Ti(C_11_H_10_N_3_)_2_(NEt_2_)_2_], was prepared by direct reaction of 2-(*N*-phenyl­methyl­imino­meth­yl)-1*H*-imidazole and [Ti(NEt_2_)_4_]. The Ti^IV^ atom is in a pseudo-octa­hedral coordination environ­ment with the imidazolido-group N-atoms occupying apical positions and amido- and imino-N-atoms *cis*-located in the equatorial plane. The presence of two bidentate chelating ligands determines the chirality of the Ti^IV^ atom. The crystallographically independent unit, except for its phenyl rings, adopts nearly pseudo-*C*_2_ symmetry (rotation around a twofold axis passing through the Ti atom and the centre of the imino-*N*⋯imino-N segment). The Ti—N_amido_, Ti—N_imidazolido_, and Ti—N_imino_ bond lengths essentially differ, increasing by approximately 0.2 Å in the series. All ligating N atoms are in a nearly planar environment, which is indicative of additional *p*π–*d*π donations towards the metal atom. The two diaza­metallacyclic units are planar within 0.03 and 0.05 Å.

## Related literature

For mononuclear neutral Ti^IV^ complexes bearing two chelating amido-imino and two amido ligands see: Xiang *et al.* (2008 ▶); Zi *et al.* (2008 ▶). For closely related mononuclear neutral Ti^IV^ complexes bearing two chelating amido-amino and two amido ligands see: Fandos *et al.* (2005 ▶); Kempe (1997 ▶); Marsh (2004 ▶); Oberthur *et al.* (1997 ▶); Smolensky *et al.* (2005 ▶); Xiang *et al.* (2008 ▶); Zaher *et al.* (2008 ▶). For the practical applications of the complexes of the type, see: McKnight & Waymouth (1998 ▶); Fix *et al.* (1990 ▶). For procedures used in the complex preparation, see: Bürger & Dämmen (1974 ▶); Bradley & Thomas (1960 ▶); Armarego & Perrin (1997 ▶). For a description of the configuration of the coordination entities, see: Connely *et al.* (2005 ▶). For a description of the Cambridge Structural Database, see: Allen (2002 ▶).
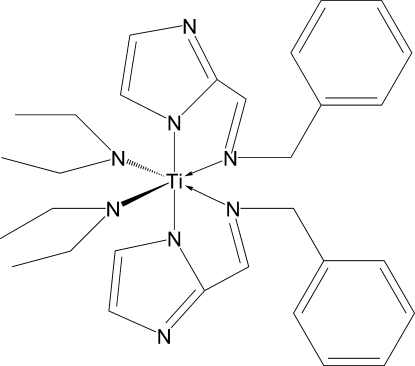


## Experimental

### 

#### Crystal data

[Ti(C_4_H_10_N)_2_(C_11_H_10_N_3_)_2_]
*M* *_r_* = 560.60
Triclinic, 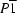

*a* = 9.6465 (9) Å
*b* = 10.3796 (10) Å
*c* = 16.3341 (16) Å
α = 102.931 (2)°
β = 102.082 (2)°
γ = 93.184 (2)°
*V* = 1549.7 (3) Å^3^
*Z* = 2
Mo *K*α radiation
μ = 0.31 mm^−1^
*T* = 296 K
0.35 × 0.24 × 0.14 mm

#### Data collection

BRUKER SMART APEXII diffractometer
Absorption correction: multi-scan (*SADABS*; Sheldrick, 1996 ▶) *T*_min_ = 0.900, *T*_max_ = 0.958
7867 measured reflections
5478 independent reflections
3387 reflections with *I* > 2σ(*I*)
*R*_int_ = 0.029

#### Refinement

*R*[*F* ^2^ > 2σ(*F* ^2^)] = 0.044
*wR*(*F* ^2^) = 0.100
*S* = 0.94
5478 reflections
356 parameters
H-atom parameters constrained
Δρ_max_ = 0.27 e Å^−3^
Δρ_min_ = −0.25 e Å^−3^

### 

Data collection: *APEX2* (Bruker, 2007 ▶); cell refinement: *SAINT-Plus* (Bruker, 2007 ▶); data reduction: *SAINT-Plus*; program(s) used to solve structure: *SHELXS97* (Sheldrick, 2008 ▶); program(s) used to refine structure: *SHELXL97* (Sheldrick, 2008 ▶); molecular graphics: *SHELXTL* (Sheldrick, 2008 ▶) and *OLEX2* (Dolomanov *et al.*, 2009 ▶); software used to prepare material for publication: *SHELXTL* and *OLEX2*.

## Supplementary Material

Crystal structure: contains datablocks I, global. DOI: 10.1107/S1600536811013183/dn2674sup1.cif

Structure factors: contains datablocks I. DOI: 10.1107/S1600536811013183/dn2674Isup2.hkl

Additional supplementary materials:  crystallographic information; 3D view; checkCIF report

## Figures and Tables

**Fig. 1.**
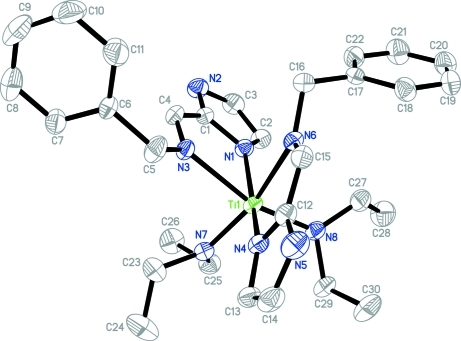
Molecular view of I with the atom labeling scheme [A- (or Λ-) enantiomer is depicted; for C/A and Δ/Λ notation see: Connely et al. (2005)]. Thermal ellipsoids are shown at the 30% probability level. All H-atoms are omitted for clarity.

## supplementary crystallographic information

### Comment

Mononuclear neutral Ti(IV) complexes bearing two chelating amido-imino and two amido ligands (Xiang *et al.*, 2008; Zi *et al.*., 2008) and closely related ones bearing two chelating amido-amino and two amido ligands (Fandos *et al.*., 2005; Kempe, 1997; Marsh, 2004; Oberthur *et al.*, 1997; Smolensky *et al.*, 2005; Xiang *et al.*, 2008; Zaher *et al.*, 2008) are known as non-metallocene components of the catalytic systems for olefin polymerization (McKnight & Waymouth, 1998) and as precursors of the compounds for metal nitride films depositions from the gas phase (Fix *et al.*, 1990). The title compound, (C_11_H_10_N_3_)_2_Ti(NEt_2_)_2_, (I), also belongs to the former family and was prepared by a direct reaction of 2-(*N*-phenylmethyliminomethyl)-1*H*-imidazole and Ti(NEt_2_)_4_ (see Experimental).

The Ti atom in I is in a pseudo-octahedral coordination environment, with imidazolido-group N-atoms occupying apical positions and amido- and imino-N-atoms *cis*-located in the equatorial plane [coordination environment *OC*-6–33 (Connely *et al.*, 2005)]. Presence of two bidentate chelating ligands determines chirality of the Ti-centre. Crystallographically independent unit of I, except of its Ph-rings, nearly adopts pseudo-*C*_2_ symmetry (rotation around a 2-fold axis passing through Ti-atom and the centre of the imino-*N*···imino-N segment). Ti—N_amido_ [1.892 (2) and 1.897 (2) Å], Ti—N_imidazolido_ [2.115 (2) and 2.1170 (19) Å], and Ti—N_imino_ [2.302 (2) and 2.302 (2) Å] bond lengths essentially differ (increase by approximately 0.2 Å in the series). All ligating N-atoms N1, N4, N3, N6, N7, N8 are in nearly planar environment [valent angles sums: 359.2 (5), 359.5 (5), 359.9 (5), 359.3 (5), 359.2 (5), and 358.7 (5)°, respectively] what is indicative of additional *p*π–*d*π donations towards the metal centre. Ti-atom lays in the imidazole rings r. m. s. planes N1/C1/N2/C3/C2 (PL1) and N4/C12/N5/C14/C13 (PL2) [deviations -0.274 (4) and 0.202 (4) Å, respectively]. Both diazametallacyclyc moieties in the molecule of I are planar within 0.03 and 0.05 Å.

Analysis of the Cambridge Structural Database [CSD; release May 2009 (Allen, 2002)] for mononuclear neutral Ti(IV) complexes bearing two chelating amido-imino and two amido ligands retrieves only 3 entries (8 fragments). These are three bis[2-(*N*-aryliminomethyl)-1*H*-pyroll-1-idyl-κ*N*^1^,*N*^1'^]bis(dimethylamido)titanium(IV) complexes (Xiang *et al.*, 2008 and Zi *et al.*., 2008). Of interest, only one of these cited complexes, [*N*,*N*'-(1,1'-binaphthalin-2,2'-diyl)bi(2-iminomethyl-1*H*- pyrrol-1-idyl)-κ*N*^1^,*N*^1^,*N*^1'^,*N*^1'^]bis(dimethylamido)titanium(IV) (Xiang *et al.*, 2008), adopts the same (*OC*-6–33) configuration as the complex I, while the other two complexes, bis(dimethylamido)bis(2-{*N*-[1-(2-methoxynaphthalin-1-yl)naphthalin-2-yl] iminomethyl}-1*H*-pyrrol-1-idyl-κ*N*^1^,*N*^1'^)- and bis(dimethylamido)bis(2-{*N*-[2-(2-methoxy-6-methylphenyl)-3- methylphenyl]iminomethyl}-1*H*-pyrrol-1-idyl-κ*N*^1^,*N*^1'^)titaniums(IV) (Zi *et al.*., 2008) exhibit (*OC*-6–1'3) configuration (pyrollyidyl moieties in *cis*- and imino-moieties in *trans*-positions). For all these three latter complexes, the observed tendencies for the Ti—N bond lengths are the same as in the case of I, with their values in I well matching the earlier reported ranges.

### Experimental

All operations were performed under argon atmosphere in conventional glassware or in all-sealed evacuated glass vessels with application of the high-vacuum line (the residual pressure of non-condensable gases within 1.5–1.0×10 ^-3^ Torr; 1 Torr = 133 Pa). Ti(NEt_2_)_4_ was prepared as described earlier (Bürger & Dämmen, 1974; Bradley & Thomas, 1960). All other chemicals were commercially available and purified by conventional methods (Armarego & Perrin, 1997). Solvents were purified by distillation over sodium benzophenoneketyl (diethyl ether, THF), Na—K alloy (toluene, benzene), and CaH_2_ (chloroform). Deuterated solvents were dried similarly. — NMR spectra were recorded on a Varian INOVA-400 instrument. For ^1^H and ^13^C spectra, the solvent [δ_H_ = 7.16 and δ_C_ = 128.00 (C_6_D_6_)] or TMS (δ_H_ = 0.00 and δ_C_ = 0.0) (CDCl_3_) resonances were used as internal reference standards. — Chromato-mass spectra were measured on Agilent 6890 Series GC system equipped with HP 5973 mass-selective detector.

2-(*N*-Phenylmethyliminomethyl)-1*H*-imidazole, II: To a solution of 1*H*-imidazole-2-carbaldehyde (1.92 g, 20.0 mmol) in methanol (20 ml), a solution of benzylamine (2.14 g, 20 mmol) in methanol (10 ml) was added dropwise under reflux and stirring during 30 min. The reaction mixture was refluxed for additional 6 h, cooled dow to ambient temperature and concentrated under reduced pressure. The formed crystalline material was collected by filtration and re-crystallized from the minimal amount of refluxing methanol what gave 3.33 g of II (90%). — ^1^H NMR (298 K, CDCl_3_): δ = 4.72 (s, 2 H, CH_2_), 7.08 (broad s, 2 H, CH in imidazole), 7.24–7.39 (m, 5 H, CH in Ph), 8.34 (s, 1 H, N═CH). — ^13^C{^1^H} NMR (298 K, CDCl_3_): δ = 64.11 (CH_2_Ph), 127.79 (*p*-CH in Ph), 127.79, 128.35 (*o*-, *m*-CH in Ph), 137.74 (C in Ph), 144.33 (CH═N), 152.79 (C in imidazole). Imidazole ring CH-carbon signals are not observed (too broad due to exchange). EI MS (70 eV) *m/z* (%): 185 (31) [*M*]^+.^, 184 (20) [*M* – H^.^]^+^, 169 (87) [*M* – H^.^ – NH^.^]^.+^, 157 (16) [*M* – HCN]^.+^, 117 (55) [[*M* – C_3_H_4_N_2_]^.+^, 91 (100) [C_7_H_7_]^+^, 81 (16) C_4_H_5_N_2_]^+^, 69 (42) [C_3_H_5_N_2_]^+^, 65 (29) [C_3_H_2_N_2_]^+^.

Complex I: To a solution of II (0.74 g, 2 mmol) in toluene (10 ml), a solution of Ti(NEt_2_)_4_ (0.67 g, 2 mmol) in toluene (10 ml) was added under stirring and cooling. The reaction mixture was heated at 353 K for 8 h. The resultant mixture was cooled down to room temperature and then left to stay at 255 K for several days. The mother liquor was decanted from the orange crystals, the crystals were washed with minimal amount of cold toluene and dried on the high-vacuum line what gave 0.73 g of I (65%). — ^1^H NMR (298 K, C_6_D_6_): δ = 0.56 (virt. t, an X-part of an AB*X*_3_ spin system, 12 H, ^3^*J*_AX_ = ^3^*J*_BX_ = 7.0 Hz, NCH_2_C*H*_3_), 3.60, 3.96 (both virt.dq, an AB-part of an AB*X*_3_ spin system, 4 H + 4H, ^3^*J*_AX_ = ^3^*J*_BX_ = 7.0 Hz, ^2^*J*_AB_ = 14.1 Hz, NC*H*_2_CH_3_), 3.86, 4.01 (AB spin system, 2 H + 2 H, 14.6 Hz, NCH_2_), 6.56, 6.99 (both m, 4 H + 6 H, CH in Ph), 7.56 (broadened s, 2 H, N═CH), 7.75, 7.81 (both broadened s, 2 H + 2 H, CH in imidazole). — ^13^C{^1^H} NMR (298 K, C_6_D_6_): δ =12.39 (CH_3_), 45.57 (CH_2_ in Et), 59.88 (CH_2_Ph), 128.79, 128.69 (*o*-, *m*-CH in Ph), 130.21 (*p*-CH in Ph), 134.06 (CH in imidazole), 142.34 (C in Ph), 152.37, 159.44 (CH═N and C in imidazole).

A crystal of (I) suitable for X-ray diffraction analysis was picked up from the isolated material and mounted inside a Lindemann glass capillary (diameter 0.5 mm; N_2_-filled glove-box).

### Refinement

H atoms were treated as riding atoms with distances C—H = 0.96 (CH_3_), 0.97 (CH_2_), 0.93 Å (C_Ar_H), and *U*_iso_(H) = 1.5 *U*_eq_(C), 1.2 *U*_eq_(C), and 1.2 *U*_eq_(C), respectively.

### Crystal data

**Table:**

[Ti(C_4_H_10_N)_2_(C_11_H_10_N_3_)_2_]	*Z* = 2
*M**_r_* = 560.60	*F*(000) = 596
Triclinic, *P*1	*D*_x_ = 1.201 Mg m^−^^3^
Hall symbol: -P 1	Mo *K*α radiation, λ = 0.71073 Å
*a* = 9.6465 (9) Å	Cell parameters from 4167 reflections
*b* = 10.3796 (10) Å	θ = 2.3–27.8°
*c* = 16.3341 (16) Å	µ = 0.31 mm^−^^1^
α = 102.931 (2)°	*T* = 296 K
β = 102.082 (2)°	Block, orange
γ = 93.184 (2)°	0.35 × 0.24 × 0.14 mm
*V* = 1549.7 (3) Å^3^	

### Data collection

**Table:**

BRUKER SMART APEXII diffractometer	5478 independent reflections
Radiation source: fine-focus sealed tube	3387 reflections with *I* > 2σ(*I*)
graphite	*R*_int_ = 0.029
Detector resolution: 8.333 pixels mm^-1^	θ_max_ = 25.1°, θ_min_ = 2.0°
phi and ω scans	*h* = −11→11
Absorption correction: multi-scan (*SADABS*; Sheldrick, 1996)	*k* = −8→12
*T*_min_ = 0.900, *T*_max_ = 0.958	*l* = −19→19
7867 measured reflections	

### Refinement

**Table:**

Refinement on *F*^2^	Primary atom site location: structure-invariant direct methods
Least-squares matrix: full	Secondary atom site location: difference Fourier map
*R*[*F*^2^ > 2σ(*F*^2^)] = 0.044	Hydrogen site location: inferred from neighbouring sites
*wR*(*F*^2^) = 0.100	H-atom parameters constrained
*S* = 0.94	*w* = 1/[σ^2^(*F*_o_^2^) + (0.0394*P*)^2^] where *P* = (*F*_o_^2^ + 2*F*_c_^2^)/3
5478 reflections	(Δ/σ)_max_ = 0.001
356 parameters	Δρ_max_ = 0.27 e Å^−^^3^
0 restraints	Δρ_min_ = −0.25 e Å^−^^3^

### Special details

**Table:**

Geometry. All e.s.d.'s (except the e.s.d. in the dihedral angle between two l.s. planes) are estimated using the full covariance matrix. The cell e.s.d.'s are taken into account individually in the estimation of e.s.d.'s in distances, angles and torsion angles; correlations between e.s.d.'s in cell parameters are only used when they are defined by crystal symmetry. An approximate (isotropic) treatment of cell e.s.d.'s is used for estimating e.s.d.'s involving l.s. planes.
Refinement. Refinement of *F*^2^ against ALL reflections. The weighted *R*-factor *wR* and goodness of fit *S* are based on *F*^2^, conventional *R*-factors *R* are based on *F*, with *F* set to zero for negative *F*^2^. The threshold expression of *F*^2^ > σ(*F*^2^) is used only for calculating *R*-factors(gt) *etc*. and is not relevant to the choice of reflections for refinement. *R*-factors based on *F*^2^ are statistically about twice as large as those based on *F*, and *R*- factors based on ALL data will be even larger.

### Fractional atomic coordinates and isotropic or equivalent isotropic displacement parameters (Å^2^)

**Table:**

	*x*	*y*	*z*	*U*_iso_*/*U*_eq_	
Ti1	0.67106 (5)	0.95033 (5)	0.77116 (3)	0.03941 (15)	
N1	0.8932 (2)	0.9452 (2)	0.81198 (12)	0.0408 (5)	
N2	1.0873 (2)	0.8371 (2)	0.79390 (15)	0.0529 (6)	
N3	0.7318 (2)	0.79631 (19)	0.66290 (12)	0.0416 (5)	
N4	0.4688 (2)	0.9508 (2)	0.69011 (12)	0.0413 (5)	
N5	0.3353 (3)	1.0264 (2)	0.58256 (15)	0.0617 (7)	
N6	0.7129 (2)	1.0613 (2)	0.67001 (13)	0.0425 (5)	
N7	0.6114 (2)	0.8134 (2)	0.81961 (13)	0.0439 (5)	
N8	0.6623 (2)	1.1057 (2)	0.85617 (12)	0.0439 (5)	
C1	0.9513 (3)	0.8501 (2)	0.76137 (16)	0.0409 (6)	
C2	1.0016 (3)	0.9955 (3)	0.88178 (16)	0.0492 (7)	
H2	0.9975	1.0629	0.9292	0.059*	
C3	1.1177 (3)	0.9302 (3)	0.87046 (19)	0.0576 (8)	
H3	1.2055	0.9469	0.9097	0.069*	
C4	0.8635 (3)	0.7761 (2)	0.68127 (16)	0.0457 (7)	
H4	0.9008	0.7145	0.6432	0.055*	
C5	0.6388 (3)	0.7312 (3)	0.57744 (17)	0.0595 (8)	
H5A	0.6090	0.7998	0.5479	0.071*	
H5B	0.5537	0.6876	0.5866	0.071*	
C6	0.7024 (3)	0.6313 (3)	0.51933 (15)	0.0424 (6)	
C7	0.6961 (3)	0.5009 (3)	0.52388 (18)	0.0561 (7)	
H7	0.6502	0.4748	0.5632	0.067*	
C8	0.7553 (4)	0.4085 (3)	0.4723 (2)	0.0782 (10)	
H8	0.7499	0.3207	0.4770	0.094*	
C9	0.8212 (4)	0.4434 (5)	0.4150 (2)	0.0871 (12)	
H9	0.8612	0.3796	0.3800	0.105*	
C10	0.8305 (4)	0.5713 (5)	0.4071 (2)	0.0901 (12)	
H10	0.8763	0.5949	0.3671	0.108*	
C11	0.7702 (4)	0.6667 (3)	0.4600 (2)	0.0723 (9)	
H11	0.7759	0.7543	0.4551	0.087*	
C12	0.4652 (3)	1.0178 (3)	0.62701 (16)	0.0447 (6)	
C13	0.3286 (3)	0.9160 (3)	0.68532 (17)	0.0506 (7)	
H13	0.2928	0.8692	0.7201	0.061*	
C14	0.2500 (3)	0.9621 (3)	0.6202 (2)	0.0618 (8)	
H14	0.1509	0.9509	0.6039	0.074*	
C15	0.5979 (3)	1.0702 (3)	0.61621 (16)	0.0482 (7)	
H15	0.6013	1.1101	0.5710	0.058*	
C16	0.8495 (3)	1.1028 (3)	0.65087 (17)	0.0505 (7)	
H16A	0.8329	1.1056	0.5907	0.061*	
H16B	0.9150	1.0369	0.6597	0.061*	
C17	0.9174 (3)	1.2367 (3)	0.70619 (16)	0.0420 (6)	
C18	0.8462 (3)	1.3481 (3)	0.70256 (18)	0.0562 (7)	
H18	0.7563	1.3397	0.6663	0.067*	
C19	0.9076 (4)	1.4714 (3)	0.7523 (2)	0.0653 (8)	
H19	0.8589	1.5456	0.7491	0.078*	
C20	1.0391 (4)	1.4856 (3)	0.8062 (2)	0.0716 (9)	
H20	1.0803	1.5691	0.8393	0.086*	
C21	1.1098 (3)	1.3760 (3)	0.8111 (2)	0.0703 (9)	
H21	1.1988	1.3847	0.8484	0.084*	
C22	1.0488 (3)	1.2524 (3)	0.76066 (19)	0.0565 (8)	
H22	1.0983	1.1786	0.7639	0.068*	
C23	0.5296 (3)	0.6855 (3)	0.77301 (19)	0.0622 (8)	
H23A	0.5953	0.6182	0.7681	0.075*	
H23B	0.4858	0.6915	0.7150	0.075*	
C24	0.4141 (3)	0.6393 (4)	0.8133 (2)	0.0974 (13)	
H24A	0.3581	0.5617	0.7745	0.146*	
H24B	0.3538	0.7087	0.8243	0.146*	
H24C	0.4570	0.6182	0.8666	0.146*	
C25	0.6712 (3)	0.8214 (3)	0.91142 (17)	0.0575 (8)	
H25A	0.5934	0.8079	0.9389	0.069*	
H25B	0.7185	0.9103	0.9385	0.069*	
C26	0.7767 (3)	0.7214 (3)	0.9281 (2)	0.0741 (9)	
H26A	0.7304	0.6328	0.9029	0.111*	
H26B	0.8106	0.7327	0.9892	0.111*	
H26C	0.8558	0.7355	0.9027	0.111*	
C27	0.7500 (3)	1.2329 (3)	0.87333 (17)	0.0583 (8)	
H27A	0.6945	1.2917	0.8445	0.070*	
H27B	0.8314	1.2174	0.8476	0.070*	
C28	0.8044 (3)	1.3036 (3)	0.96745 (19)	0.0813 (10)	
H28A	0.8729	1.3772	0.9724	0.122*	
H28B	0.8485	1.2428	0.9986	0.122*	
H28C	0.7261	1.3356	0.9910	0.122*	
C29	0.5402 (3)	1.1148 (3)	0.89731 (18)	0.0582 (8)	
H29A	0.5757	1.1442	0.9593	0.070*	
H29B	0.4895	1.0268	0.8850	0.070*	
C30	0.4360 (3)	1.2086 (3)	0.8683 (2)	0.0849 (11)	
H30A	0.4079	1.1864	0.8064	0.127*	
H30B	0.4807	1.2984	0.8884	0.127*	
H30C	0.3533	1.2005	0.8916	0.127*	

### Atomic displacement parameters (Å^2^)

**Table:**

	*U*^11^	*U*^22^	*U*^33^	*U*^12^	*U*^13^	*U*^23^
Ti1	0.0373 (3)	0.0441 (3)	0.0362 (3)	0.0076 (2)	0.0114 (2)	0.0048 (2)
N1	0.0366 (11)	0.0433 (13)	0.0401 (12)	0.0043 (10)	0.0085 (10)	0.0058 (10)
N2	0.0379 (13)	0.0595 (16)	0.0599 (15)	0.0086 (11)	0.0096 (11)	0.0125 (12)
N3	0.0420 (13)	0.0385 (13)	0.0404 (12)	0.0061 (10)	0.0092 (10)	0.0012 (9)
N4	0.0366 (12)	0.0443 (13)	0.0416 (12)	0.0087 (10)	0.0103 (10)	0.0051 (10)
N5	0.0516 (15)	0.0729 (18)	0.0581 (16)	0.0194 (13)	0.0036 (13)	0.0159 (13)
N6	0.0431 (13)	0.0416 (13)	0.0435 (13)	0.0068 (10)	0.0166 (11)	0.0049 (10)
N7	0.0416 (12)	0.0482 (14)	0.0434 (13)	0.0079 (10)	0.0128 (10)	0.0102 (10)
N8	0.0448 (13)	0.0472 (14)	0.0386 (12)	0.0076 (11)	0.0141 (10)	0.0032 (10)
C1	0.0391 (15)	0.0396 (16)	0.0455 (15)	0.0054 (12)	0.0145 (12)	0.0086 (12)
C2	0.0430 (16)	0.0553 (18)	0.0435 (15)	0.0028 (14)	0.0054 (13)	0.0047 (13)
C3	0.0396 (16)	0.067 (2)	0.0599 (19)	0.0002 (15)	−0.0002 (14)	0.0154 (16)
C4	0.0463 (16)	0.0411 (16)	0.0513 (16)	0.0121 (13)	0.0190 (13)	0.0052 (13)
C5	0.0564 (18)	0.059 (2)	0.0504 (17)	0.0120 (15)	0.0041 (14)	−0.0076 (14)
C6	0.0495 (16)	0.0390 (17)	0.0353 (14)	0.0104 (13)	0.0079 (12)	0.0022 (12)
C7	0.068 (2)	0.0449 (19)	0.0528 (17)	0.0056 (15)	0.0137 (15)	0.0063 (14)
C8	0.085 (3)	0.053 (2)	0.083 (3)	0.0220 (19)	0.003 (2)	−0.0005 (19)
C9	0.072 (2)	0.100 (3)	0.073 (3)	0.029 (2)	0.019 (2)	−0.018 (2)
C10	0.093 (3)	0.118 (4)	0.064 (2)	0.001 (3)	0.042 (2)	0.009 (2)
C11	0.098 (3)	0.061 (2)	0.062 (2)	0.0069 (19)	0.0242 (19)	0.0191 (17)
C12	0.0457 (16)	0.0457 (17)	0.0404 (15)	0.0101 (13)	0.0075 (13)	0.0063 (13)
C13	0.0393 (16)	0.0533 (18)	0.0575 (18)	0.0065 (14)	0.0144 (14)	0.0063 (14)
C14	0.0370 (16)	0.070 (2)	0.071 (2)	0.0150 (15)	0.0053 (15)	0.0059 (17)
C15	0.0602 (18)	0.0472 (17)	0.0401 (15)	0.0106 (14)	0.0136 (14)	0.0133 (13)
C16	0.0518 (17)	0.0512 (18)	0.0540 (17)	0.0053 (14)	0.0225 (14)	0.0140 (14)
C17	0.0475 (16)	0.0415 (17)	0.0442 (15)	0.0051 (13)	0.0197 (13)	0.0167 (12)
C18	0.0552 (18)	0.054 (2)	0.0625 (19)	0.0080 (15)	0.0092 (15)	0.0237 (15)
C19	0.076 (2)	0.047 (2)	0.081 (2)	0.0136 (17)	0.0242 (19)	0.0242 (17)
C20	0.083 (2)	0.051 (2)	0.077 (2)	−0.0070 (19)	0.027 (2)	0.0038 (17)
C21	0.057 (2)	0.067 (2)	0.076 (2)	−0.0044 (17)	−0.0002 (16)	0.0124 (18)
C22	0.0504 (17)	0.0522 (19)	0.072 (2)	0.0127 (15)	0.0154 (15)	0.0210 (16)
C23	0.0602 (19)	0.059 (2)	0.066 (2)	−0.0031 (16)	0.0068 (16)	0.0202 (16)
C24	0.071 (2)	0.107 (3)	0.117 (3)	−0.022 (2)	0.011 (2)	0.052 (3)
C25	0.0617 (19)	0.063 (2)	0.0530 (18)	0.0099 (15)	0.0175 (15)	0.0194 (15)
C26	0.071 (2)	0.082 (2)	0.071 (2)	0.0200 (19)	0.0022 (17)	0.0321 (18)
C27	0.069 (2)	0.0525 (19)	0.0522 (17)	0.0082 (16)	0.0216 (15)	0.0034 (14)
C28	0.086 (2)	0.076 (2)	0.067 (2)	−0.0043 (19)	0.0150 (18)	−0.0107 (17)
C29	0.0580 (18)	0.060 (2)	0.0598 (18)	0.0188 (15)	0.0255 (15)	0.0065 (15)
C30	0.068 (2)	0.092 (3)	0.096 (3)	0.039 (2)	0.0227 (19)	0.012 (2)

### Geometric parameters (Å, °)

**Table:**

Ti1—N7	1.892 (2)	C13—C14	1.366 (4)
Ti1—N8	1.897 (2)	C13—H13	0.9300
Ti1—N1	2.115 (2)	C14—H14	0.9300
Ti1—N4	2.117 (2)	C15—H15	0.9300
Ti1—N3	2.302 (2)	C16—C17	1.505 (3)
Ti1—N6	2.302 (2)	C16—H16A	0.9700
N1—C2	1.356 (3)	C16—H16B	0.9700
N1—C1	1.365 (3)	C17—C22	1.366 (3)
N2—C1	1.335 (3)	C17—C18	1.384 (3)
N2—C3	1.363 (3)	C18—C19	1.377 (4)
N3—C4	1.283 (3)	C18—H18	0.9300
N3—C5	1.482 (3)	C19—C20	1.363 (4)
N4—C12	1.362 (3)	C19—H19	0.9300
N4—C13	1.362 (3)	C20—C21	1.367 (4)
N5—C12	1.329 (3)	C20—H20	0.9300
N5—C14	1.354 (3)	C21—C22	1.382 (4)
N6—C15	1.284 (3)	C21—H21	0.9300
N6—C16	1.480 (3)	C22—H22	0.9300
N7—C23	1.465 (3)	C23—C24	1.514 (4)
N7—C25	1.471 (3)	C23—H23A	0.9700
N8—C27	1.466 (3)	C23—H23B	0.9700
N8—C29	1.471 (3)	C24—H24A	0.9600
C1—C4	1.426 (3)	C24—H24B	0.9600
C2—C3	1.365 (4)	C24—H24C	0.9600
C2—H2	0.9300	C25—C26	1.523 (4)
C3—H3	0.9300	C25—H25A	0.9700
C4—H4	0.9300	C25—H25B	0.9700
C5—C6	1.492 (3)	C26—H26A	0.9600
C5—H5A	0.9700	C26—H26B	0.9600
C5—H5B	0.9700	C26—H26C	0.9600
C6—C7	1.370 (3)	C27—C28	1.514 (4)
C6—C11	1.379 (4)	C27—H27A	0.9700
C7—C8	1.363 (4)	C27—H27B	0.9700
C7—H7	0.9300	C28—H28A	0.9600
C8—C9	1.337 (5)	C28—H28B	0.9600
C8—H8	0.9300	C28—H28C	0.9600
C9—C10	1.362 (5)	C29—C30	1.516 (4)
C9—H9	0.9300	C29—H29A	0.9700
C10—C11	1.398 (5)	C29—H29B	0.9700
C10—H10	0.9300	C30—H30A	0.9600
C11—H11	0.9300	C30—H30B	0.9600
C12—C15	1.423 (4)	C30—H30C	0.9600
			
N7—Ti1—N8	102.26 (9)	N5—C14—H14	124.4
N7—Ti1—N1	97.40 (8)	C13—C14—H14	124.4
N8—Ti1—N1	95.28 (8)	N6—C15—C12	118.9 (2)
N7—Ti1—N4	94.93 (8)	N6—C15—H15	120.6
N8—Ti1—N4	96.73 (8)	C12—C15—H15	120.6
N1—Ti1—N4	160.55 (8)	N6—C16—C17	112.8 (2)
N7—Ti1—N3	90.78 (8)	N6—C16—H16A	109.0
N8—Ti1—N3	164.61 (8)	C17—C16—H16A	109.0
N1—Ti1—N3	74.71 (7)	N6—C16—H16B	109.0
N4—Ti1—N3	90.18 (7)	C17—C16—H16B	109.0
N7—Ti1—N6	160.41 (8)	H16A—C16—H16B	107.8
N8—Ti1—N6	95.38 (8)	C22—C17—C18	118.2 (2)
N1—Ti1—N6	89.31 (7)	C22—C17—C16	121.9 (2)
N4—Ti1—N6	74.39 (7)	C18—C17—C16	119.9 (2)
N3—Ti1—N6	73.21 (7)	C19—C18—C17	120.5 (3)
C2—N1—C1	103.6 (2)	C19—C18—H18	119.8
C2—N1—Ti1	139.84 (18)	C17—C18—H18	119.8
C1—N1—Ti1	115.71 (15)	C20—C19—C18	120.6 (3)
C1—N2—C3	102.6 (2)	C20—C19—H19	119.7
C4—N3—C5	120.8 (2)	C18—C19—H19	119.7
C4—N3—Ti1	112.44 (16)	C19—C20—C21	119.5 (3)
C5—N3—Ti1	126.65 (16)	C19—C20—H20	120.3
C12—N4—C13	103.6 (2)	C21—C20—H20	120.3
C12—N4—Ti1	116.54 (16)	C20—C21—C22	120.0 (3)
C13—N4—Ti1	139.36 (18)	C20—C21—H21	120.0
C12—N5—C14	102.8 (2)	C22—C21—H21	120.0
C15—N6—C16	117.2 (2)	C17—C22—C21	121.2 (3)
C15—N6—Ti1	112.47 (17)	C17—C22—H22	119.4
C16—N6—Ti1	129.59 (16)	C21—C22—H22	119.4
C23—N7—C25	113.6 (2)	N7—C23—C24	115.6 (3)
C23—N7—Ti1	126.96 (17)	N7—C23—H23A	108.4
C25—N7—Ti1	118.60 (17)	C24—C23—H23A	108.4
C27—N8—C29	113.4 (2)	N7—C23—H23B	108.4
C27—N8—Ti1	125.74 (16)	C24—C23—H23B	108.4
C29—N8—Ti1	119.51 (17)	H23A—C23—H23B	107.4
N2—C1—N1	114.7 (2)	C23—C24—H24A	109.5
N2—C1—C4	127.1 (2)	C23—C24—H24B	109.5
N1—C1—C4	118.2 (2)	H24A—C24—H24B	109.5
N1—C2—C3	108.3 (2)	C23—C24—H24C	109.5
N1—C2—H2	125.9	H24A—C24—H24C	109.5
C3—C2—H2	125.9	H24B—C24—H24C	109.5
N2—C3—C2	110.8 (2)	N7—C25—C26	114.5 (2)
N2—C3—H3	124.6	N7—C25—H25A	108.6
C2—C3—H3	124.6	C26—C25—H25A	108.6
N3—C4—C1	118.5 (2)	N7—C25—H25B	108.6
N3—C4—H4	120.8	C26—C25—H25B	108.6
C1—C4—H4	120.8	H25A—C25—H25B	107.6
N3—C5—C6	116.3 (2)	C25—C26—H26A	109.5
N3—C5—H5A	108.2	C25—C26—H26B	109.5
C6—C5—H5A	108.2	H26A—C26—H26B	109.5
N3—C5—H5B	108.2	C25—C26—H26C	109.5
C6—C5—H5B	108.2	H26A—C26—H26C	109.5
H5A—C5—H5B	107.4	H26B—C26—H26C	109.5
C7—C6—C11	117.8 (2)	N8—C27—C28	115.8 (2)
C7—C6—C5	120.8 (2)	N8—C27—H27A	108.3
C11—C6—C5	121.4 (3)	C28—C27—H27A	108.3
C8—C7—C6	121.7 (3)	N8—C27—H27B	108.3
C8—C7—H7	119.1	C28—C27—H27B	108.3
C6—C7—H7	119.1	H27A—C27—H27B	107.4
C9—C8—C7	120.2 (3)	C27—C28—H28A	109.5
C9—C8—H8	119.9	C27—C28—H28B	109.5
C7—C8—H8	119.9	H28A—C28—H28B	109.5
C8—C9—C10	120.8 (3)	C27—C28—H28C	109.5
C8—C9—H9	119.6	H28A—C28—H28C	109.5
C10—C9—H9	119.6	H28B—C28—H28C	109.5
C9—C10—C11	119.3 (3)	N8—C29—C30	114.2 (2)
C9—C10—H10	120.4	N8—C29—H29A	108.7
C11—C10—H10	120.4	C30—C29—H29A	108.7
C6—C11—C10	120.2 (3)	N8—C29—H29B	108.7
C6—C11—H11	119.9	C30—C29—H29B	108.7
C10—C11—H11	119.9	H29A—C29—H29B	107.6
N5—C12—N4	114.9 (2)	C29—C30—H30A	109.5
N5—C12—C15	127.7 (3)	C29—C30—H30B	109.5
N4—C12—C15	117.5 (2)	H30A—C30—H30B	109.5
N4—C13—C14	107.7 (2)	C29—C30—H30C	109.5
N4—C13—H13	126.2	H30A—C30—H30C	109.5
C14—C13—H13	126.2	H30B—C30—H30C	109.5
N5—C14—C13	111.1 (2)		
			
N7—Ti1—N1—C2	−84.9 (3)	C3—N2—C1—N1	0.1 (3)
N8—Ti1—N1—C2	18.2 (3)	C3—N2—C1—C4	−179.8 (3)
N4—Ti1—N1—C2	146.2 (3)	C2—N1—C1—N2	−0.1 (3)
N3—Ti1—N1—C2	−173.7 (3)	Ti1—N1—C1—N2	−171.77 (16)
N6—Ti1—N1—C2	113.5 (3)	C2—N1—C1—C4	179.8 (2)
N7—Ti1—N1—C1	82.46 (18)	Ti1—N1—C1—C4	8.1 (3)
N8—Ti1—N1—C1	−174.42 (17)	C1—N1—C2—C3	0.1 (3)
N4—Ti1—N1—C1	−46.4 (3)	Ti1—N1—C2—C3	168.4 (2)
N3—Ti1—N1—C1	−6.32 (16)	C1—N2—C3—C2	0.0 (3)
N6—Ti1—N1—C1	−79.08 (17)	N1—C2—C3—N2	0.0 (3)
N7—Ti1—N3—C4	−93.32 (18)	C5—N3—C4—C1	174.6 (2)
N8—Ti1—N3—C4	54.8 (4)	Ti1—N3—C4—C1	−1.4 (3)
N1—Ti1—N3—C4	4.14 (17)	N2—C1—C4—N3	175.6 (2)
N4—Ti1—N3—C4	171.75 (18)	N1—C1—C4—N3	−4.3 (4)
N6—Ti1—N3—C4	98.12 (18)	C4—N3—C5—C6	6.5 (4)
N7—Ti1—N3—C5	91.0 (2)	Ti1—N3—C5—C6	−178.21 (17)
N8—Ti1—N3—C5	−120.8 (3)	N3—C5—C6—C7	87.1 (3)
N1—Ti1—N3—C5	−171.5 (2)	N3—C5—C6—C11	−92.4 (3)
N4—Ti1—N3—C5	−3.9 (2)	C11—C6—C7—C8	0.4 (4)
N6—Ti1—N3—C5	−77.5 (2)	C5—C6—C7—C8	−179.0 (3)
N7—Ti1—N4—C12	−166.58 (17)	C6—C7—C8—C9	−0.3 (5)
N8—Ti1—N4—C12	90.43 (18)	C7—C8—C9—C10	0.1 (6)
N1—Ti1—N4—C12	−37.4 (3)	C8—C9—C10—C11	0.1 (6)
N3—Ti1—N4—C12	−75.79 (17)	C7—C6—C11—C10	−0.3 (4)
N6—Ti1—N4—C12	−3.30 (16)	C5—C6—C11—C10	179.2 (3)
N7—Ti1—N4—C13	23.5 (3)	C9—C10—C11—C6	0.0 (5)
N8—Ti1—N4—C13	−79.5 (3)	C14—N5—C12—N4	0.7 (3)
N1—Ti1—N4—C13	152.7 (3)	C14—N5—C12—C15	−179.1 (3)
N3—Ti1—N4—C13	114.3 (3)	C13—N4—C12—N5	−0.8 (3)
N6—Ti1—N4—C13	−173.2 (3)	Ti1—N4—C12—N5	−174.10 (17)
N7—Ti1—N6—C15	59.2 (3)	C13—N4—C12—C15	179.0 (2)
N8—Ti1—N6—C15	−95.02 (18)	Ti1—N4—C12—C15	5.7 (3)
N1—Ti1—N6—C15	169.74 (18)	C12—N4—C13—C14	0.5 (3)
N4—Ti1—N6—C15	0.48 (17)	Ti1—N4—C13—C14	171.3 (2)
N3—Ti1—N6—C15	95.51 (18)	C12—N5—C14—C13	−0.4 (3)
N7—Ti1—N6—C16	−110.1 (3)	N4—C13—C14—N5	−0.1 (3)
N8—Ti1—N6—C16	95.6 (2)	C16—N6—C15—C12	173.1 (2)
N1—Ti1—N6—C16	0.4 (2)	Ti1—N6—C15—C12	2.3 (3)
N4—Ti1—N6—C16	−168.9 (2)	N5—C12—C15—N6	174.4 (3)
N3—Ti1—N6—C16	−73.9 (2)	N4—C12—C15—N6	−5.4 (4)
N8—Ti1—N7—C23	146.5 (2)	C15—N6—C16—C17	104.0 (3)
N1—Ti1—N7—C23	−116.4 (2)	Ti1—N6—C16—C17	−87.1 (3)
N4—Ti1—N7—C23	48.5 (2)	N6—C16—C17—C22	119.2 (3)
N3—Ti1—N7—C23	−41.8 (2)	N6—C16—C17—C18	−60.9 (3)
N6—Ti1—N7—C23	−7.2 (4)	C22—C17—C18—C19	0.5 (4)
N8—Ti1—N7—C25	−45.04 (19)	C16—C17—C18—C19	−179.5 (3)
N1—Ti1—N7—C25	52.04 (19)	C17—C18—C19—C20	−0.3 (5)
N4—Ti1—N7—C25	−143.04 (18)	C18—C19—C20—C21	−0.5 (5)
N3—Ti1—N7—C25	126.72 (18)	C19—C20—C21—C22	1.0 (5)
N6—Ti1—N7—C25	161.2 (2)	C18—C17—C22—C21	0.1 (4)
N7—Ti1—N8—C27	151.9 (2)	C16—C17—C22—C21	−180.0 (3)
N1—Ti1—N8—C27	53.1 (2)	C20—C21—C22—C17	−0.9 (5)
N4—Ti1—N8—C27	−111.6 (2)	C25—N7—C23—C24	52.2 (3)
N3—Ti1—N8—C27	4.6 (4)	Ti1—N7—C23—C24	−138.8 (2)
N6—Ti1—N8—C27	−36.7 (2)	C23—N7—C25—C26	61.8 (3)
N7—Ti1—N8—C29	−42.4 (2)	Ti1—N7—C25—C26	−108.1 (2)
N1—Ti1—N8—C29	−141.14 (18)	C29—N8—C27—C28	54.1 (3)
N4—Ti1—N8—C29	54.19 (19)	Ti1—N8—C27—C28	−139.3 (2)
N3—Ti1—N8—C29	170.3 (3)	C27—N8—C29—C30	60.6 (3)
N6—Ti1—N8—C29	129.06 (18)	Ti1—N8—C29—C30	−106.9 (3)
